# Adoption of COVID-19 Contact Tracing Apps: A Balance Between Privacy and Effectiveness

**DOI:** 10.2196/25726

**Published:** 2021-03-04

**Authors:** Emily Seto, Priyanka Challa, Patrick Ware

**Affiliations:** 1 Institute of Health Policy, Management and Evaluation Dalla Lana School of Public Health University of Toronto Toronto, ON Canada; 2 Centre for Global eHealth Innovation University Health Network Toronto, ON Canada; 3 Techna Institute University Health Network Toronto, ON Canada

**Keywords:** mobile apps, COVID-19, contact tracing, exposure notification, privacy, effectiveness, app, surveillance, tracing, transmission, security, digital health

## Abstract

With the relative ubiquity of smartphones, contact tracing and exposure notification apps have been looked to as novel methods to help reduce the transmission of COVID-19. Many countries have created apps that lie across a spectrum from privacy-first approaches to those that have very few privacy measures. The level of privacy incorporated into an app is largely based on the societal norms and values of a particular country. Digital health technologies can be highly effective and preserve privacy at the same time, but in the case of contact tracing and exposure notification apps, there is a trade-off between increased privacy measures and the effectiveness of the app. In this article, examples from various countries are used to highlight how characteristics of contract tracing and exposure notification apps contribute to the perceived levels of privacy awarded to citizens and how this impacts an app’s effectiveness. We conclude that finding the right balance between privacy and effectiveness, while critical, is challenging because it is highly context-specific.

## Introduction

Many countries around the world have released contact tracing and exposure notification apps in an attempt to help combat the spread of COVID-19 [1,2]. However, the technologies used, adoption rates, and potential impact of the apps have been extremely varied across countries. Moreover, each country has developed contact tracing apps that meet the level of privacy required for their citizens. Often, increased privacy has been deemed a fair trade-off for a decrease in the potential effectiveness of the app.

## Privacy-Related Characteristics of COVID-19 Apps

Although privacy laws provide a foundation that can inform the design and implementation of exposure notification and true contact tracing apps [3], it is the types of technologies used (eg, quick response [QR] codes, GPS, Bluetooth Low Energy) and the way they are applied within those legal frameworks that determine the level of privacy afforded to citizens. The important distinction between contact tracing apps and exposure notification apps is that the former collects tracking data so that public health authorities can determine who individuals have been in contact with, as well as the location and time of the contact. On the other hand, exposure notification apps collect only the data required to determine if an individual may have been in close contact with someone who has been identified as being positive for COVID-19, which provides significantly more privacy. Table 1 expands upon research by Liu and Guo [4], which was published in the initial months of the pandemic. Specifically, it presents privacy-related characteristics of COVID-19 apps in various countries. The examples have been selected to demonstrate a spectrum of privacy-related features in countries where information about their apps is publicly available, as well as to demonstrate that the optimal balance between privacy and effectiveness may be culturally dependent.

**Table 1:** Privacy characteristics of COVID-19 contact tracing and exposure notification apps.

Country	App name (month of launch)	Voluntary or mandatory	Technology	App data bound by privacy laws	Consent for data sharing required	Centralized or decentralized data storage	Approximate adoption rate, % (month of reporting)
Australia	COVIDSafe (April 2020) [5]	Voluntary	Bluetooth [5]	Yes	Yes	Centralized	21.6 (July 2020) [6]
China	HealthCode (February 2020) [7]	Voluntary (required to move around cities)	May use GPS or records of individual’s location	Yes	No	Information not found	~64 (April 2020)^a^ [8]
Canada	COVID Alert (July 2020) [9]	Voluntary	Bluetooth	Yes	Yes	Decentralized	~15 (December 2020)^a,b^ [10]
Germany	Corona-Warn-App (June 2020) [11]	Voluntary	Bluetooth	Yes	Yes	Centralized (pseudonymized contact identifiers)	1.4 (July 2020) [6]
Hong Kong	StayHomeSafe (April 2020) [12]	Mandatory for 14-day home quarantine	Bluetooth and geofencing technology using wristbands	Yes	Yes	Decentralized	Information not found
Hong Kong	LeaveHomeSafe (November 2020) [12]	Voluntary	QR^c^ codes	Yes	Yes	Decentralized	Information not found
New Zealand	NZ COVID Tracer App (May 2020) [13]	Voluntary	Bluetooth, QR codes, location alert through push notifications	Yes	Yes	Decentralized	10.7 (July 2020) [6]
Russia	Social Monitoring (April 2020) [14]	Mandatory for individuals with COVID-19; voluntary for others	App seeks consent to access Bluetooth, GPS, and camera	Yes	Yes	Centralized	Information not found
Singapore	TraceTogether (March 2020) [15]	Voluntary (required to move around the city)	Bluetooth	Yes	Not for infected persons	Centralized	70 (December 2020) [16]
South Korea	Self-Quarantine Safety Protection (March 2020) [17]	Required for new arrivals for 2 weeks (telephone calls are alternative option)	Bluetooth, GPS, credit card transactions, surveillance cameras, and others	Yes	Yes	Centralized	Information not found
South Korea	Corona 100m (February 2020) [18]	Voluntary	GPS, uses data from public government sources	Yes	No	Decentralized	~2 (end of February 2020, 3 weeks after rollout)^a^ [18]
Saudi Arabia	Tawakkalna (May 2020) [19]	Voluntary	GPS	Information not found	Information not found	Centralized	4.9 (July 2020) [6]
United Kingdom	NHS COVID-19 (September 2020) [20]	Voluntary	Bluetooth 4.0 or higher	Yes	Yes	Centralized (nonidentifiable information)	40 (October 2020) [21]

^a^Estimated adoption rate based on number of downloads divided by the country’s entire population.

^b^In Canada, the app was rolled out in Ontario first; as of December 2020, only 9 of the 13 provinces and territories had adopted the app.

^c^QR: quick response.

## Privacy Measures Related to Adoption

Beyond the inherent value of privacy as a human right and as a protection against bias and stigma, the intent of increasing an app’s privacy measures is to broaden its adoption. This has been confirmed by studies conducted since the start of the pandemic, which have demonstrated the important role of trust and perceived privacy in influencing the adoption and use of COVID-19 apps [22-24]. The perception of privacy is not only determined by a user’s interpretation of existing safeguards (eg, the underlying technology, whether the app is voluntary, and the degree of data centralization), but also requires those details be effectively communicated to citizens, which is not always the case. For example, an analysis of COVID-19 app privacy policies concluded that improvements to the readability of privacy policies could lead to increased usage [25].

Modelling by a research group at Oxford has indicated that the pandemic can be stopped if about 60% of the population uses the app. Lower adoption rates of 15% would still reduce infection and deaths by about 8% and 6%, respectively [26]. At the time of writing, Singapore and China have been able to reach the 60% threshold, while many other countries have reported adoption rates greater than 15%, including Ireland, Canada (ie, Ontario, where the app was first launched), Germany, and Iceland (the first country in Europe to launch their app) [27].

While adoption rates are affected by numerous nuanced factors, they are likely highly influenced by three broad categories of privacy-related factors highlighted in Table 1. First, apps that are mandatory would presumably result in higher adoption rates than voluntary apps. For example, although use of the apps in Singapore and China is technically voluntary, the apps are required for citizens to move around freely in cities, which may have resulted in the high adoption rates of those apps. Specifically, citizens in China are unable to move freely within cities and enter establishments without showing their color-coded individual QR codes at checkpoints. A color code (green, yellow, or red) is assigned depending on the user’s travel history and health status, with green allowing unrestricted movement, and yellow and red indicating different quarantine requirements [28].

Second, the technology employed in the apps will largely dictate how intrusive the apps are to an individual’s privacy. Public transparency about the technology used may also increase confidence in the use of the app. To increase adoption, some countries—including Canada, Germany, and the United Kingdom—have opted to use the well-documented and highly vetted Google and Apple Exposure Notification application programming interface (API), which enables the swapping of anonymous identifier beacons (ie, random strings of numbers that are frequently changed) between phones in close proximity via Bluetooth Low Energy [29]. This provides a very high level of privacy because no identifiable data is transmitted. Other countries, such as China, have opted for technology that tracks the location of individuals (eg, GPS), which may be a deterrent to their use. Local Chinese governments have developed their own apps with algorithms that assign the color code, but little information has been made available on the details of how the algorithms work [28]. As another example, in South Korea, all people coming into the country are required to quarantine for two weeks and to download the “Self-Quarantine Safety Protection” app, which tracks a person’s movement via GPS to monitor compliance with isolation procedures (telephone calls are an option if someone does not have a smartphone) [30]. After the two-week quarantine period, the app tells the user that they are able to delete the app from their phones. In addition, Corona 100m is a voluntary app in South Korea that was built by a private developer after the government made certain data about patients with COVID-19 freely available [31]. The Corona 100m app shows the location of people infected with the virus, the date the infection was confirmed, and the nationality, sex, and age of the infected person. Alerts are sent to users when they are within 100 meters of the latest tracked location visited by someone positive for COVID-19. Data used by the app comes from smartphone location logs, credit and debit card transactions, and an extensive network of surveillance cameras [32].

Third, data governance in terms of privacy laws related to the app data, user consent for data sharing, and centralization of data storage are also important factors that can impact citizens’ comfort level with using the apps. Countries with data governance laws and policies that protect privacy, such as the United Kingdom and Canada, appear to have relatively good adoption rates. On the other hand, a lower adoption rate was reported in Saudi Arabia, where it is not clear if the apps are bound by privacy laws and whether consent for data sharing is required. Some countries have centralized the storage of data into a database controlled by a public health authority, while others have decentralized data storage (ie, data is stored only on an individual’s smartphone). While a centralized data storage system could provide added value through the ability to analyze the data for trends and adoption information, decentralized systems may invoke more trust in the app, which could drive up adoption. Germany’s exposure notification app was initially developed to support a centralized approach, but was met with much criticism, leading to a change to a decentralized model [33].

## The Privacy Versus Effectiveness Trade-Off

The trade-off between privacy and effectiveness is apparent at both the individual and system level. For example, apps leveraging the Google and Apple Exposure Notification API, which was designed with a privacy-first approach, do not provide users with the identity of the person with COVID-19 that they were in close proximity to, or information about the location or time of the potential exposure beyond the fact that it was within the past 14 days. Therefore, the user is provided with little context to determine the actual risk (eg, whether personal protective equipment was used) and how long they should be exercising extra precaution to reduce the risk of transmission of the virus.

The limited collection of data and decentralized systems that are used to protect privacy also hinder the ability of governments to analyze aggregate data, including demographics, time stamps, and geolocalization, which could inform the design and implementation of more targeted public health strategies. Specifically, COVID-19 apps that prioritize effectiveness over privacy could aggregate reliable demographic information about potentially exposed users that is grounded in a specific place and time. In its absence, governments and public health officials must largely rely on information gathered when patients positive for COVID-19 interact with the health system (which is almost always temporally dissociated from the time of infection) or human-driven contact tracing, which relies on citizens’ recall and willingness to report accurate information. While useful, these data may be less reliable and comprehensive compared to data collected via a COVID-19 app, thus limiting the data’s ability to inform the types of targeted interventions that could simultaneously decrease the spread of COVID-19 and avoid the negative societal consequences of more generalized lockdowns.

Furthermore, the lack of detailed and centralized data limits evaluations of the effectiveness of these apps. For instance, many exposure notification systems will only be able to determine the number of downloads and the number of users who have chosen to identify themselves as positive for COVID-19 through the app. It is not known whether the user has since deleted the app or has chosen to turn off Bluetooth. Other unknowns include how many people have been notified of a potential exposure, how many people chose to be tested because of an alert, and how many people tested positive for COVID-19 earlier than they would have otherwise due to an alert. In these situations, there is mainly anecdotal effectiveness evidence of users getting alerted about an exposure, getting tested, and then modifying their behavior to reduce the transmission of the virus. Evidence of the effectiveness of the app can help drive adoption of the app, as well as inform future implementations of and improvements to the app. Furthermore, the perception that the app is effective may incentivize individuals to download and use the app, which in itself would presumably increase its effectiveness.

## Other Factors Impacting the Adoption of COVID-19 Apps

In addition to privacy concerns, a barrier to adoption is the inability for some to download the app. While the proportion of people who own smartphones is high and increasing (upwards of 80% in some countries), there is still a considerable number of people who do not own smartphones [34]. In addition, a criticism of using the Google and Apple Exposure Notification API is that it works only on phones that were released in the past five years or so, which could have the effect of excluding lower-income communities that may have particularly high rates of COVID-19 transmission [35]. Singapore’s innovative solution to help reach citizens that are unable to download the TraceTogether app was to distribute a device called the TraceTogether token, which works by swapping identifier beacons via Bluetooth, similar to the app [36].

There are other methods that have been used to try to increase adoption of voluntary exposure notification apps. One is to incorporate features in the app to increase its perceived value [37]. For example, some exposure notification apps can provide COVID-19 test results. In the United Kingdom, the NHS COVID-19 app has features such as ordering COVID-19 tests, receiving test results, regional risk score alerts, symptom recorders, and a self-isolation countdown and advice [38].

A second way to increase adoption of these apps is through social influence and media campaigns. In Canada, influential brands and high-profile individuals like athletes have partnered to promote the use of the COVID Alert app [39]. New Zealand has used humor and creativity in their efforts to inform citizens about the NZ COVID Tracer app, and the pandemic more generally, through comedic skits with well-known television personalities and a call for filmmakers to submit short videos [40]. Opportunities to communicate the privacy safeguards as well as personal and societal benefits should also be explored in these campaigns.

Finally, reducing user effort by making the app easier to download and use may also increase adoption. For example, during the launch of the COVID Alert app in Ontario, Canada, a government alert was sent to smartphones regarding the app, with information on how to download the app, which runs in the background after setup without further user interaction.

## Conclusion

With increased privacy, there are inherent trade-offs in the effectiveness of COVID-19 contact tracing and exposure notification apps. The effectiveness of the apps might be impossible to evaluate fully due to the lack of collected data, especially for apps with privacy-first approaches, as well as confounding factors like community lockdowns. However, given the assumption that higher adoption translates into increased effectiveness, broadening adoption of voluntary apps is a goal of many countries, which can be achieved through several techniques. These include investing in a promotional campaign that may involve hiring a professional marketing firm, partnering with high-profile personalities to endorse the app, and increasing ease of app download via smartphone alerts by the government that link to the app. While the level of privacy required for a COVID-19 contact tracing and exposure notification app will depend on factors including whether it is voluntary, the underlying technology, and degree of data centralization, translation of those important safeguards into a user’s perception of privacy will occur within the context of the norms and values of their country. Therefore, striking the right balance between privacy and effectiveness requires careful consideration, especially as the urgency to reduce transmission of the virus evolves based on fluctuating case numbers and vaccination efforts.

## Abbreviations

API: application programming interface
QR: quick response
